# 4-[Bis(4-fluoro­phen­yl)meth­yl]piperazin-1-ium bis­(trichloro­acetate) 0.4-hydrate

**DOI:** 10.1107/S1600536812009282

**Published:** 2012-03-07

**Authors:** A. S. Dayananda, H. S. Yathirajan, Ulrich Flörke

**Affiliations:** a Department of Studies in Chemistry, University of Mysore, Manasagangotri, Mysore 570 006, India; b Department Chemie, Fakultät für Naturwissenschaften, Universität Paderborn, Warburgerstr. 100, D-33098 Paderborn, Germany

## Abstract

The title compound, C_17_H_20_F_2_N_2_^2+^·2C_2_Cl_3_O_2_^−^·0.4H_2_O, has twofold protonated N atoms. The trichloro­acetate anions each show one disordered Cl atom with site occupation factors of 0.945 (7):0.055 (7) 0.945 (8):0.055 (8). In the crystal, N—H⋯O, O(water)—H⋯O and O(water)—H⋯F inter­actions connect the components into a three-dimensional network.

## Related literature

For the biological activity of piperazines, see: Bogatcheva *et al.* (2006 ▶); Brockunier *et al.* (2004 ▶). For a review pharmacological and toxicological information for piperazine derivatives, see: Elliott, (2011 ▶). For related structures, see: Betz *et al.* (2011 ▶, 2011*a* ▶); Perpétuo & Janczak (2006 ▶). For graph-set analysis of hydrogen bonds, see: Bernstein *et al.* (1995 ▶); Etter *et al.* (1990 ▶). For puckering analysis, see: Cremer & Pople (1975 ▶).
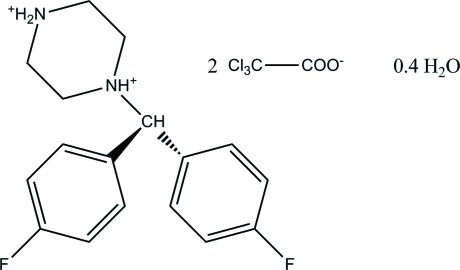


## Experimental

### 

#### Crystal data

C_17_H_20_F_2_N_2_^2+^·2C_2_Cl_3_O_2_^−^·0.4H_2_O
*M* *_r_* = 622.30
Monoclinic, 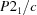

*a* = 8.8101 (7) Å
*b* = 33.555 (3) Å
*c* = 9.4453 (7) Å
β = 108.723 (2)°
*V* = 2644.5 (3) Å^3^
*Z* = 4
Mo *K*α radiation
μ = 0.70 mm^−1^
*T* = 130 K
0.43 × 0.25 × 0.23 mm

#### Data collection

Bruker SMART APEX diffractometer
Absorption correction: multi-scan (*SADABS*; Sheldrick, 2004 ▶) *T*_min_ = 0.754, *T*_max_ = 0.856
25025 measured reflections
6309 independent reflections
5475 reflections with *I* > 2σ(*I*)
*R*_int_ = 0.033

#### Refinement

*R*[*F* ^2^ > 2σ(*F* ^2^)] = 0.040
*wR*(*F* ^2^) = 0.094
*S* = 1.12
6309 reflections
343 parameters
5 restraints
H atoms treated by a mixture of independent and constrained refinement
Δρ_max_ = 0.44 e Å^−3^
Δρ_min_ = −0.33 e Å^−3^

### 

Data collection: *SMART* (Bruker, 2002 ▶); cell refinement: *SAINT* (Bruker, 2002 ▶); data reduction: *SAINT*; program(s) used to solve structure: *SHELXTL* (Sheldrick, 2008 ▶); program(s) used to refine structure: *SHELXTL*; molecular graphics: *SHELXTL*; software used to prepare material for publication: *SHELXTL* and local programs.

## Supplementary Material

Crystal structure: contains datablock(s) I, global. DOI: 10.1107/S1600536812009282/zj2061sup1.cif

Structure factors: contains datablock(s) I. DOI: 10.1107/S1600536812009282/zj2061Isup2.hkl

Supplementary material file. DOI: 10.1107/S1600536812009282/zj2061Isup3.cml

Additional supplementary materials:  crystallographic information; 3D view; checkCIF report

## Figures and Tables

**Table 1:** Hydrogen-bond geometry (Å, °)

*D*—H⋯*A*	*D*—H	H⋯*A*	*D*⋯*A*	*D*—H⋯*A*
N1—H1*B*⋯O3^i^	0.93	1.75	2.6756 (19)	172
N2—H2*A*⋯O4^ii^	0.92	1.88	2.7735 (19)	162
N2—H2*B*⋯O2^iii^	0.92	1.84	2.7487 (19)	168
O100—H102⋯F2^iv^	0.84 (1)	2.32 (8)	2.878 (4)	125 (8)
O100—H101⋯O1	0.84 (1)	1.94 (4)	2.733 (4)	158 (9)

Symmetry codes: (i) 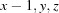; (ii) 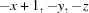; (iii) 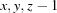; (iv) 
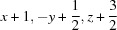
.

**Fig. 1.**
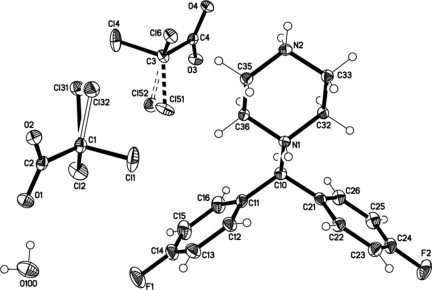
Molecular structure with displacement ellipsoids drawn at the 50% probability level.

**Fig. 2.**
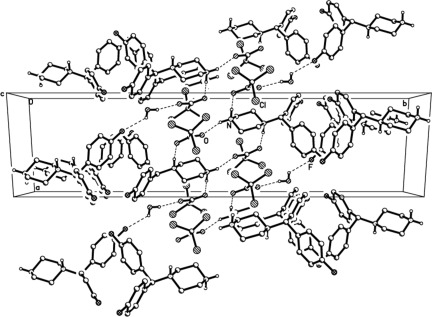
Crystal packing viewed along [001] with H bonding as dotted lines. Hydrogen atoms not involved are omitted.

## supplementary crystallographic information

### Comment

A review on the current pharmacological and toxicological information for piperazine derivatives is described (Elliott, 2011). 4,4'-Difluorobenzhydryl piperazine is an intermediate for the preparation of flunarizine which is a calcium channel blocker. Piperazines are among the most important building blocks in today's drug discovery and are found in biologically active compounds across a number of different therapeutic areas (Brockunier *et al.*, 2004; Bogatcheva *et al.*, 2006). Recently, we have reported the crystal structures of 4-[bis(4-fluorophenyl)methyl]piperazin-1-ium 2-(2-phenylethyl)benzoate (Betz *et al.*, 2011) and 4-[bis(4-fluorophenyl) methyl]piperazin-1-ium picrate (Betz *et al.*, 2011*a*). The crystal structures of melaminium bis(trifluoroacetate) trihydrate and melaminium bis(trichloroacetate) dihydrate have been reported (Perpétuo & Janczak, 2006). In the course of our studies on the salts of piperazines and in view of the importance of piperazines, we now report the crystal and molecular structure of the title salt. The presence of water in the crystal may be due to moisture from trichloroacetic acid. The water is part of intermolecular N—H···O, O(water)—H···O and O(water)—H···F interactions of the crystal packing (Figure 2 and Table 1). The molecular structure exhibits no unusual geometries. Both fluoro-phenyl rings form a dihedral angle of 68.64 (6)°, and each one chloro ligand of the two anions is disordered over two positions Cl31/Cl32 and Cl51/Cl52 with 0.945 (8)/0.055 (8) occupation.

### Experimental

4,4'-Difluorobenzhydryl piperazine was obtained from *R. L*. Fine Chem., Bengaluru, India. 4,4'-Difluorobenzhydryl piperazine (2.88 g, 0.01 mol) and trichloroacetic acid (1.63 g, 0.01 mol) were dissolved in hot ethanol solution and stirred over a heating magnetic stirrer for few minutes (330 K). The resulting solution was allowed to cool slowly at room temperature. X-ray quality crystals of the title compound were obtained by the slow evaporation of ethanol (m.p.: 388–393 K).

### Refinement

H atoms were clearly identified in difference syntheses, idealized and refined riding on the carbon atoms with C—H = 0.95 (aromatic) - 1.00 Å, N—H = 0.92 - 0.93 Å and with isotropic displacement parameters *U*_iso_(H) = 1.2U(C/N_eq_). Water H atoms were refined freely with *DFIX* O—H 0.84 (1) and *DFIX* H···H 1.4 (1) Å.

### Crystal data

**Table:**

C_17_H_20_F_2_N_2_^2+^·2C_2_Cl_3_O_2_^−^·0.4H_2_O	*F*(000) = 1264
*M**_r_* = 622.30	*D*_x_ = 1.563 Mg m^−^^3^
Monoclinic, *P*2_1_/*c*	Mo *K*α radiation, λ = 0.71073 Å
Hall symbol: -P 2ybc	Cell parameters from 6364 reflections
*a* = 8.8101 (7) Å	θ = 2.4–28.1°
*b* = 33.555 (3) Å	µ = 0.70 mm^−^^1^
*c* = 9.4453 (7) Å	*T* = 130 K
β = 108.723 (2)°	Block, colourless
*V* = 2644.5 (3) Å^3^	0.43 × 0.25 × 0.23 mm
*Z* = 4	

### Data collection

**Table:**

Bruker SMART APEX diffractometer	6309 independent reflections
Radiation source: sealed tube	5475 reflections with *I* > 2σ(*I*)
Graphite monochromator	*R*_int_ = 0.033
φ and ω scans	θ_max_ = 27.9°, θ_min_ = 2.4°
Absorption correction: multi-scan (*SADABS*; Sheldrick, 2004)	*h* = −11→11
*T*_min_ = 0.754, *T*_max_ = 0.856	*k* = −44→35
25025 measured reflections	*l* = −12→12

### Refinement

**Table:**

Refinement on *F*^2^	Primary atom site location: structure-invariant direct methods
Least-squares matrix: full	Secondary atom site location: difference Fourier map
*R*[*F*^2^ > 2σ(*F*^2^)] = 0.040	Hydrogen site location: inferred from neighbouring sites
*wR*(*F*^2^) = 0.094	H atoms treated by a mixture of independent and constrained refinement
*S* = 1.12	*w* = 1/[σ^2^(*F*_o_^2^) + (0.0376*P*)^2^ + 1.238*P*] where *P* = (*F*_o_^2^ + 2*F*_c_^2^)/3
6309 reflections	(Δ/σ)_max_ < 0.001
343 parameters	Δρ_max_ = 0.44 e Å^−^^3^
5 restraints	Δρ_min_ = −0.33 e Å^−^^3^

### Special details

**Table:**

Geometry. All e.s.d.'s (except the e.s.d. in the dihedral angle between two l.s. planes) are estimated using the full covariance matrix. The cell e.s.d.'s are taken into account individually in the estimation of e.s.d.'s in distances, angles and torsion angles; correlations between e.s.d.'s in cell parameters are only used when they are defined by crystal symmetry. An approximate (isotropic) treatment of cell e.s.d.'s is used for estimating e.s.d.'s involving l.s. planes.
Refinement. Refinement of *F*^2^ against ALL reflections. The weighted *R*-factor *wR* and goodness of fit *S* are based on *F*^2^, conventional *R*-factors *R* are based on *F*, with *F* set to zero for negative *F*^2^. The threshold expression of *F*^2^ > σ(*F*^2^) is used only for calculating *R*-factors(gt) *etc*. and is not relevant to the choice of reflections for refinement. *R*-factors based on *F*^2^ are statistically about twice as large as those based on *F*, and *R*- factors based on ALL data will be even larger.

### Fractional atomic coordinates and isotropic or equivalent isotropic displacement parameters (Å^2^)

**Table:**

	*x*	*y*	*z*	*U*_iso_*/*U*_eq_	Occ. (<1)
Cl1	0.42896 (7)	0.08738 (2)	0.57017 (6)	0.04709 (17)	
Cl2	0.73291 (7)	0.122760 (16)	0.73174 (6)	0.03824 (14)	
Cl31	0.7180 (2)	0.039699 (19)	0.66377 (16)	0.0288 (3)	0.945 (7)
Cl32	0.655 (3)	0.0375 (3)	0.625 (2)	0.030 (3)*	0.055 (7)
O1	0.57723 (16)	0.09907 (4)	0.94355 (15)	0.0257 (3)	
O2	0.51880 (15)	0.03603 (4)	0.86531 (14)	0.0218 (3)	
C1	0.6146 (2)	0.07955 (6)	0.71377 (19)	0.0210 (4)	
C2	0.5687 (2)	0.07066 (5)	0.85888 (19)	0.0183 (3)	
Cl4	1.04646 (6)	0.05481 (2)	0.43723 (6)	0.04426 (16)	
Cl51	0.7926 (3)	0.11414 (3)	0.3445 (2)	0.0353 (4)	0.945 (9)
Cl52	0.852 (3)	0.1118 (3)	0.385 (2)	0.030 (4)*	0.055 (9)
Cl6	0.72048 (5)	0.031251 (14)	0.28437 (5)	0.02344 (11)	
O3	0.97280 (16)	0.10468 (4)	0.13520 (15)	0.0243 (3)	
O4	0.89212 (15)	0.04212 (4)	0.06973 (14)	0.0210 (3)	
C3	0.8647 (2)	0.06867 (6)	0.29606 (19)	0.0209 (4)	
C4	0.9108 (2)	0.07214 (5)	0.14910 (19)	0.0175 (3)	
F1	0.47404 (19)	0.21857 (4)	0.64013 (15)	0.0531 (4)	
F2	−0.14523 (17)	0.26032 (4)	−0.34231 (15)	0.0443 (3)	
N1	0.22837 (17)	0.11350 (4)	0.04351 (15)	0.0163 (3)	
H1B	0.1356	0.1125	0.0701	0.020*	
N2	0.26897 (17)	0.03018 (4)	−0.02096 (16)	0.0176 (3)	
H2A	0.2369	0.0040	−0.0286	0.021*	
H2B	0.3617	0.0318	−0.0457	0.021*	
C10	0.2875 (2)	0.15637 (5)	0.05572 (19)	0.0193 (4)	
H1A	0.3867	0.1565	0.0260	0.023*	
C11	0.3345 (2)	0.17165 (5)	0.2157 (2)	0.0224 (4)	
C12	0.2363 (3)	0.16824 (6)	0.3048 (2)	0.0265 (4)	
H12A	0.1357	0.1551	0.2669	0.032*	
C13	0.2841 (3)	0.18384 (6)	0.4487 (2)	0.0328 (5)	
H13A	0.2180	0.1812	0.5103	0.039*	
C14	0.4278 (3)	0.20306 (6)	0.4998 (2)	0.0353 (5)	
C15	0.5268 (3)	0.20753 (7)	0.4159 (2)	0.0399 (5)	
H15A	0.6257	0.2213	0.4543	0.048*	
C16	0.4798 (3)	0.19138 (6)	0.2723 (2)	0.0325 (5)	
H16A	0.5480	0.1939	0.2127	0.039*	
C21	0.1666 (2)	0.18308 (5)	−0.05342 (19)	0.0205 (4)	
C22	0.0095 (2)	0.18688 (6)	−0.0532 (2)	0.0242 (4)	
H22A	−0.0256	0.1716	0.0151	0.029*	
C23	−0.0966 (2)	0.21262 (6)	−0.1512 (2)	0.0282 (4)	
H23A	−0.2040	0.2152	−0.1513	0.034*	
C24	−0.0419 (3)	0.23440 (6)	−0.2483 (2)	0.0294 (4)	
C25	0.1114 (3)	0.23131 (6)	−0.2544 (2)	0.0321 (5)	
H25A	0.1449	0.2466	−0.3237	0.039*	
C26	0.2160 (2)	0.20519 (6)	−0.1560 (2)	0.0270 (4)	
H26A	0.3223	0.2023	−0.1586	0.032*	
C32	0.1905 (2)	0.09844 (5)	−0.11400 (18)	0.0185 (3)	
H32A	0.2858	0.1017	−0.1468	0.022*	
H32B	0.1023	0.1145	−0.1812	0.022*	
C33	0.1419 (2)	0.05501 (5)	−0.12607 (19)	0.0192 (4)	
H33A	0.0407	0.0520	−0.1028	0.023*	
H33B	0.1230	0.0457	−0.2297	0.023*	
C35	0.3000 (2)	0.04431 (5)	0.13512 (19)	0.0179 (3)	
H35A	0.3842	0.0276	0.2045	0.022*	
H35B	0.2012	0.0418	0.1628	0.022*	
C36	0.3534 (2)	0.08726 (5)	0.14877 (18)	0.0175 (3)	
H36A	0.3733	0.0964	0.2528	0.021*	
H36B	0.4551	0.0895	0.1258	0.021*	
O100	0.6383 (5)	0.17843 (13)	0.9984 (5)	0.0411 (15)	0.398 (5)
H101	0.636 (11)	0.1549 (10)	0.968 (10)	0.10 (3)*	0.398 (5)
H102	0.720 (7)	0.181 (3)	1.075 (6)	0.10 (3)*	0.398 (5)

### Atomic displacement parameters (Å^2^)

**Table:**

	*U*^11^	*U*^22^	*U*^33^	*U*^12^	*U*^13^	*U*^23^
Cl1	0.0302 (3)	0.0767 (5)	0.0258 (3)	0.0066 (3)	−0.0029 (2)	0.0160 (3)
Cl2	0.0497 (3)	0.0321 (3)	0.0420 (3)	−0.0160 (2)	0.0275 (3)	−0.0024 (2)
Cl31	0.0361 (8)	0.0300 (3)	0.0275 (5)	0.0028 (3)	0.0201 (5)	0.0011 (2)
O1	0.0276 (7)	0.0258 (7)	0.0278 (7)	−0.0033 (6)	0.0146 (6)	−0.0041 (6)
O2	0.0204 (6)	0.0216 (7)	0.0260 (7)	−0.0008 (5)	0.0112 (5)	0.0021 (5)
C1	0.0198 (9)	0.0241 (9)	0.0185 (8)	−0.0009 (7)	0.0054 (7)	0.0033 (7)
C2	0.0113 (8)	0.0248 (9)	0.0187 (8)	0.0028 (7)	0.0046 (6)	0.0042 (7)
Cl4	0.0244 (3)	0.0773 (5)	0.0237 (3)	−0.0059 (3)	−0.0027 (2)	0.0090 (3)
Cl51	0.0431 (9)	0.0314 (3)	0.0402 (7)	−0.0065 (4)	0.0258 (7)	−0.0164 (3)
Cl6	0.0226 (2)	0.0282 (2)	0.0228 (2)	−0.00551 (18)	0.01187 (17)	−0.00174 (17)
O3	0.0247 (7)	0.0210 (7)	0.0316 (7)	−0.0053 (5)	0.0150 (6)	−0.0038 (5)
O4	0.0218 (6)	0.0214 (7)	0.0223 (6)	−0.0044 (5)	0.0105 (5)	−0.0046 (5)
C3	0.0182 (9)	0.0271 (10)	0.0181 (8)	−0.0036 (7)	0.0066 (7)	−0.0044 (7)
C4	0.0114 (8)	0.0224 (9)	0.0190 (8)	0.0009 (6)	0.0054 (6)	0.0009 (7)
F1	0.0701 (10)	0.0504 (9)	0.0271 (7)	−0.0105 (7)	−0.0008 (7)	−0.0165 (6)
F2	0.0456 (8)	0.0331 (7)	0.0419 (8)	0.0057 (6)	−0.0030 (6)	0.0182 (6)
N1	0.0174 (7)	0.0155 (7)	0.0165 (7)	−0.0002 (6)	0.0064 (6)	0.0003 (5)
N2	0.0179 (7)	0.0153 (7)	0.0208 (7)	−0.0008 (6)	0.0079 (6)	−0.0005 (6)
C10	0.0200 (9)	0.0169 (9)	0.0204 (8)	−0.0036 (7)	0.0056 (7)	0.0006 (7)
C11	0.0269 (10)	0.0155 (9)	0.0202 (9)	−0.0002 (7)	0.0010 (7)	0.0013 (7)
C12	0.0321 (11)	0.0215 (10)	0.0237 (9)	−0.0024 (8)	0.0056 (8)	−0.0034 (7)
C13	0.0456 (13)	0.0256 (11)	0.0258 (10)	0.0017 (9)	0.0093 (9)	−0.0031 (8)
C14	0.0500 (14)	0.0236 (10)	0.0224 (10)	−0.0005 (9)	−0.0022 (9)	−0.0050 (8)
C15	0.0400 (13)	0.0356 (13)	0.0322 (11)	−0.0140 (10)	−0.0048 (10)	−0.0046 (9)
C16	0.0317 (11)	0.0310 (11)	0.0302 (11)	−0.0079 (9)	0.0036 (9)	0.0012 (8)
C21	0.0254 (9)	0.0156 (9)	0.0185 (8)	−0.0034 (7)	0.0042 (7)	−0.0020 (7)
C22	0.0277 (10)	0.0205 (9)	0.0238 (9)	0.0005 (8)	0.0072 (8)	0.0033 (7)
C23	0.0282 (10)	0.0241 (10)	0.0288 (10)	0.0016 (8)	0.0042 (8)	0.0002 (8)
C24	0.0374 (12)	0.0177 (9)	0.0244 (9)	0.0003 (8)	−0.0023 (8)	0.0039 (7)
C25	0.0421 (12)	0.0237 (10)	0.0282 (10)	−0.0063 (9)	0.0082 (9)	0.0074 (8)
C26	0.0306 (11)	0.0232 (10)	0.0257 (10)	−0.0050 (8)	0.0068 (8)	0.0029 (8)
C32	0.0201 (9)	0.0208 (9)	0.0144 (8)	0.0016 (7)	0.0053 (7)	−0.0001 (6)
C33	0.0171 (8)	0.0212 (9)	0.0184 (8)	0.0002 (7)	0.0047 (7)	−0.0010 (7)
C35	0.0179 (8)	0.0189 (9)	0.0183 (8)	0.0011 (7)	0.0076 (7)	0.0006 (6)
C36	0.0176 (8)	0.0183 (9)	0.0158 (8)	0.0019 (7)	0.0041 (7)	0.0011 (6)
O100	0.045 (3)	0.034 (2)	0.051 (3)	−0.0121 (19)	0.025 (2)	−0.0149 (19)

### Geometric parameters (Å, º)

**Table:**

Cl1—C1	1.7777 (19)	C12—C13	1.390 (3)
Cl2—C1	1.7616 (19)	C12—H12A	0.9500
Cl31—Cl32	0.56 (3)	C13—C14	1.364 (3)
Cl31—C1	1.766 (2)	C13—H13A	0.9500
Cl32—C1	1.733 (11)	C14—C15	1.362 (3)
O1—C2	1.231 (2)	C15—C16	1.394 (3)
O2—C2	1.251 (2)	C15—H15A	0.9500
C1—C2	1.577 (2)	C16—H16A	0.9500
Cl4—C3	1.7852 (19)	C21—C22	1.391 (3)
Cl51—Cl52	0.55 (3)	C21—C26	1.396 (3)
Cl51—C3	1.768 (2)	C22—C23	1.386 (3)
Cl52—C3	1.696 (11)	C22—H22A	0.9500
Cl6—C3	1.7646 (19)	C23—C24	1.375 (3)
O3—C4	1.246 (2)	C23—H23A	0.9500
O4—C4	1.235 (2)	C24—C25	1.373 (3)
C3—C4	1.571 (2)	C25—C26	1.389 (3)
F1—C14	1.359 (2)	C25—H25A	0.9500
F2—C24	1.362 (2)	C26—H26A	0.9500
N1—C32	1.504 (2)	C32—C33	1.513 (2)
N1—C36	1.508 (2)	C32—H32A	0.9900
N1—C10	1.522 (2)	C32—H32B	0.9900
N1—H1B	0.9300	C33—H33A	0.9900
N2—C35	1.488 (2)	C33—H33B	0.9900
N2—C33	1.490 (2)	C35—C36	1.508 (2)
N2—H2A	0.9200	C35—H35A	0.9900
N2—H2B	0.9200	C35—H35B	0.9900
C10—C21	1.515 (2)	C36—H36A	0.9900
C10—C11	1.522 (2)	C36—H36B	0.9900
C10—H1A	1.0000	O100—H101	0.840 (10)
C11—C16	1.387 (3)	O100—H102	0.839 (10)
C11—C12	1.392 (3)		
			
Cl32—Cl31—C1	77.4 (11)	C14—C13—H13A	120.7
Cl31—Cl32—C1	84.0 (13)	C12—C13—H13A	120.7
C2—C1—Cl32	114.5 (4)	F1—C14—C15	118.6 (2)
C2—C1—Cl2	112.39 (12)	F1—C14—C13	118.6 (2)
Cl32—C1—Cl2	120.7 (7)	C15—C14—C13	122.8 (2)
C2—C1—Cl31	112.80 (12)	C14—C15—C16	118.5 (2)
Cl32—C1—Cl31	18.5 (9)	C14—C15—H15A	120.7
Cl2—C1—Cl31	108.00 (11)	C16—C15—H15A	120.7
C2—C1—Cl1	105.13 (12)	C11—C16—C15	120.7 (2)
Cl32—C1—Cl1	91.9 (10)	C11—C16—H16A	119.7
Cl2—C1—Cl1	108.84 (10)	C15—C16—H16A	119.7
Cl31—C1—Cl1	109.59 (12)	C22—C21—C26	118.96 (17)
O1—C2—O2	129.59 (16)	C22—C21—C10	122.39 (16)
O1—C2—C1	116.02 (16)	C26—C21—C10	118.64 (17)
O2—C2—C1	114.21 (15)	C23—C22—C21	120.93 (18)
Cl52—Cl51—C3	73.5 (12)	C23—C22—H22A	119.5
Cl51—Cl52—C3	88.5 (16)	C21—C22—H22A	119.5
C4—C3—Cl52	116.9 (5)	C24—C23—C22	118.10 (19)
C4—C3—Cl6	112.51 (12)	C24—C23—H23A	120.9
Cl52—C3—Cl6	118.4 (7)	C22—C23—H23A	120.9
C4—C3—Cl51	112.38 (13)	F2—C24—C25	118.79 (18)
Cl52—C3—Cl51	18.0 (9)	F2—C24—C23	118.0 (2)
Cl6—C3—Cl51	108.59 (11)	C25—C24—C23	123.19 (18)
C4—C3—Cl4	104.78 (12)	C24—C25—C26	117.99 (19)
Cl52—C3—Cl4	92.2 (10)	C24—C25—H25A	121.0
Cl6—C3—Cl4	108.46 (10)	C26—C25—H25A	121.0
Cl51—C3—Cl4	110.00 (13)	C25—C26—C21	120.81 (19)
O4—C4—O3	128.92 (16)	C25—C26—H26A	119.6
O4—C4—C3	117.01 (15)	C21—C26—H26A	119.6
O3—C4—C3	113.85 (15)	N1—C32—C33	111.45 (14)
C32—N1—C36	109.84 (13)	N1—C32—H32A	109.3
C32—N1—C10	110.76 (13)	C33—C32—H32A	109.3
C36—N1—C10	110.02 (13)	N1—C32—H32B	109.3
C32—N1—H1B	108.7	C33—C32—H32B	109.3
C36—N1—H1B	108.7	H32A—C32—H32B	108.0
C10—N1—H1B	108.7	N2—C33—C32	110.77 (14)
C35—N2—C33	110.00 (13)	N2—C33—H33A	109.5
C35—N2—H2A	109.7	C32—C33—H33A	109.5
C33—N2—H2A	109.7	N2—C33—H33B	109.5
C35—N2—H2B	109.7	C32—C33—H33B	109.5
C33—N2—H2B	109.7	H33A—C33—H33B	108.1
H2A—N2—H2B	108.2	N2—C35—C36	110.01 (13)
C21—C10—N1	111.04 (14)	N2—C35—H35A	109.7
C21—C10—C11	112.61 (15)	C36—C35—H35A	109.7
N1—C10—C11	111.79 (14)	N2—C35—H35B	109.7
C21—C10—H1A	107.0	C36—C35—H35B	109.7
N1—C10—H1A	107.0	H35A—C35—H35B	108.2
C11—C10—H1A	107.0	N1—C36—C35	111.11 (14)
C16—C11—C12	118.84 (18)	N1—C36—H36A	109.4
C16—C11—C10	117.85 (18)	C35—C36—H36A	109.4
C12—C11—C10	123.26 (17)	N1—C36—H36B	109.4
C13—C12—C11	120.55 (19)	C35—C36—H36B	109.4
C13—C12—H12A	119.7	H36A—C36—H36B	108.0
C11—C12—H12A	119.7	H101—O100—H102	107 (7)
C14—C13—C12	118.6 (2)		
			
Cl31—Cl32—C1—C2	−88.9 (13)	C21—C10—C11—C12	75.9 (2)
Cl31—Cl32—C1—Cl2	50.3 (14)	N1—C10—C11—C12	−50.0 (2)
Cl31—Cl32—C1—Cl1	163.5 (12)	C16—C11—C12—C13	−0.9 (3)
Cl32—Cl31—C1—C2	99.2 (13)	C10—C11—C12—C13	−178.09 (18)
Cl32—Cl31—C1—Cl2	−135.9 (13)	C11—C12—C13—C14	1.0 (3)
Cl32—Cl31—C1—Cl1	−17.5 (13)	C12—C13—C14—F1	179.54 (19)
Cl32—C1—C2—O1	163.1 (11)	C12—C13—C14—C15	−0.2 (3)
Cl2—C1—C2—O1	20.5 (2)	F1—C14—C15—C16	179.6 (2)
Cl31—C1—C2—O1	142.94 (16)	C13—C14—C15—C16	−0.7 (4)
Cl1—C1—C2—O1	−97.70 (16)	C12—C11—C16—C15	0.0 (3)
Cl32—C1—C2—O2	−21.3 (11)	C10—C11—C16—C15	177.33 (19)
Cl2—C1—C2—O2	−163.85 (13)	C14—C15—C16—C11	0.8 (3)
Cl31—C1—C2—O2	−41.5 (2)	N1—C10—C21—C22	57.6 (2)
Cl1—C1—C2—O2	77.91 (16)	C11—C10—C21—C22	−68.6 (2)
Cl51—Cl52—C3—C4	79.5 (17)	N1—C10—C21—C26	−123.58 (17)
Cl51—Cl52—C3—Cl6	−60.3 (15)	C11—C10—C21—C26	110.19 (19)
Cl51—Cl52—C3—Cl4	−172.7 (14)	C26—C21—C22—C23	−1.0 (3)
Cl52—Cl51—C3—C4	−108.6 (16)	C10—C21—C22—C23	177.77 (17)
Cl52—Cl51—C3—Cl6	126.3 (15)	C21—C22—C23—C24	−0.2 (3)
Cl52—Cl51—C3—Cl4	7.8 (15)	C22—C23—C24—F2	−178.57 (17)
Cl52—C3—C4—O4	−167.2 (11)	C22—C23—C24—C25	1.2 (3)
Cl6—C3—C4—O4	−25.1 (2)	F2—C24—C25—C26	178.97 (18)
Cl51—C3—C4—O4	−148.05 (16)	C23—C24—C25—C26	−0.8 (3)
Cl4—C3—C4—O4	92.53 (16)	C24—C25—C26—C21	−0.6 (3)
Cl52—C3—C4—O3	17.7 (12)	C22—C21—C26—C25	1.4 (3)
Cl6—C3—C4—O3	159.81 (13)	C10—C21—C26—C25	−177.41 (17)
Cl51—C3—C4—O3	36.9 (2)	C36—N1—C32—C33	54.54 (18)
Cl4—C3—C4—O3	−82.56 (16)	C10—N1—C32—C33	176.28 (14)
C32—N1—C10—C21	56.15 (18)	C35—N2—C33—C32	58.39 (18)
C36—N1—C10—C21	177.78 (14)	N1—C32—C33—N2	−56.38 (18)
C32—N1—C10—C11	−177.16 (14)	C33—N2—C35—C36	−59.54 (17)
C36—N1—C10—C11	−55.53 (18)	C32—N1—C36—C35	−55.92 (17)
C21—C10—C11—C16	−101.3 (2)	C10—N1—C36—C35	−178.09 (13)
N1—C10—C11—C16	132.84 (18)	N2—C35—C36—N1	58.83 (18)

### Hydrogen-bond geometry (Å, º)

**Table:**

*D*—H···*A*	*D*—H	H···*A*	*D*···*A*	*D*—H···*A*
N1—H1*B*···O3^i^	0.93	1.75	2.6756 (19)	172
N2—H2*A*···O4^ii^	0.92	1.88	2.7735 (19)	162
N2—H2*B*···O2^iii^	0.92	1.84	2.7487 (19)	168
O100—H102···F2^iv^	0.84 (1)	2.32 (8)	2.878 (4)	125 (8)
O100—H101···O1	0.84 (1)	1.94 (4)	2.733 (4)	158 (9)

Symmetry codes: (i) *x*−1, *y*, *z*; (ii) −*x*+1, −*y*, −*z*; (iii) *x*, *y*, *z*−1; (iv) *x*+1, −*y*+1/2, *z*+3/2.
